# Barriers and Facilitators to the Implementation and Scale-Up of the Baby-Friendly Hospital Initiative: A Qualitative Study in Saskatchewan, Canada

**DOI:** 10.3390/healthcare14162662

**Published:** 2026-08-21

**Authors:** Shela Akbar Ali Hirani, Natasha C. Taylor

**Affiliations:** Faculty of Nursing, University of Regina, 3737 Wascana Parkway, Regina, SK S4S 0A2, Canada

**Keywords:** breastfeeding, Baby-Friendly Hospital Initiatives, healthcare settings, mothers, infants, maternity care, support

## Abstract

**Highlights:**

**What are the main findings?**

- This study identified five interconnected domains influencing BFHI implementation in Saskatchewan, Canada, including leadership and governance, workforce capacity, continuity of care, maternal psychosocial experiences, and structural determinants of breastfeeding support.
- Successful BFHI scale-up was facilitated by strong leadership, standardized breastfeeding practices, skilled providers, interdisciplinary collaboration, and community-based services, while barriers included workforce shortages, fragmented care pathways, inequities in access, and limited system monitoring.

**What are the implications of the main findings?**

- Sustainable BFHI implementation requires coordinated health system strategies that extend beyond individual provider training to strengthen leadership, workforce capacity, accountability, and integrated maternity care pathways.
- Embedding BFHI principles into routine healthcare systems can enhance equitable access to breastfeeding support and improve experiences and outcomes for diverse families.

**Abstract:**

**Background:** The Baby-Friendly Hospital Initiative (BFHI), developed by the World Health Organization (WHO) and UNICEF, provides an evidence-based framework to protect, promote, and support breastfeeding through implementation of the Ten Steps to Successful Breastfeeding. Despite its global adoption, implementation and scale-up of BFHI remain inconsistent across healthcare systems. In Saskatchewan, Canada, only one healthcare facility currently holds BFHI designation, highlighting the need to understand the health system factors influencing implementation. This study aimed to explore the barriers and facilitators influencing the implementation and scale-up of BFHI within Saskatchewan healthcare settings. **Methods:** A qualitative descriptive study was conducted using Bronfenbrenner’s socioecological model as a guiding theoretical framework. Semi-structured interviews were conducted with 26 mothers and healthcare professionals with breastfeeding-related experiences in Saskatchewan, including 11 mothers (42.3%), 6 mothers who were also healthcare professionals (23.1%), and 9 healthcare professionals without maternal experience (34.6%). Participants were recruited using purposive and maximum variation sampling. Guided by a socioecological framework, interview data were manually analyzed using an iterative thematic analysis approach. Themes were interpreted across individual, interpersonal, organizational, community, and health system levels. **Results:** Five interrelated themes characterized BFHI implementation and scale-up in Saskatchewan, including (1) leadership and governance as foundations for BFHI implementation; (2) workforce capacity, competency, and practice consistency as determinants of care delivery; (3) continuity of breastfeeding support across care settings; (4) breastfeeding as a psychosocially embedded maternal experience; and (5) structural and societal factors shaping BFHI scale-up. While supportive leadership, provincial breastfeeding guidelines, skilled healthcare providers, interdisciplinary collaboration, and community-based services facilitated BFHI-aligned care, implementation was challenged by workforce shortages, inconsistent clinical practices, limited access to specialized breastfeeding support, early postpartum discharge, geographic inequities, fragmented care pathways, and inadequate system-level monitoring. **Conclusions:** In Saskatchewan, BFHI implementation and scale-up are shaped by interconnected organizational, workforce, maternal, and structural factors. The findings provide context-specific qualitative evidence to inform future BFHI planning and implementation. Further research is needed to assess the feasibility, sustainability, and effectiveness of strategies to support BFHI scale-up across diverse healthcare and geographic settings.

## 1. Introduction

Breastfeeding is widely recognized as an important public health intervention, providing nutritional, developmental, immunological, and economic benefits for infants, mothers, and families [1,2,3]. The World Health Organization (WHO) recommends exclusive breastfeeding for the first six months of life, followed by continued breastfeeding alongside complementary foods up to two years and beyond [4]. Despite these recommendations and the well-established benefits of breastfeeding, optimal breastfeeding practices remain suboptimal globally, highlighting the need for effective health system strategies that support breastfeeding initiation, exclusivity, and continuation [4,5].

To address this need, the World Health Organization (WHO) and UNICEF launched the Baby-Friendly Hospital Initiative (BFHI) in 1991 following the Innocenti Declaration [6]. The BFHI promotes the implementation of the “Ten Steps to Successful Breastfeeding” within maternity and newborn care settings as a global standard for protecting, promoting, and supporting breastfeeding [6,7]. Evidence indicates that BFHI implementation is associated with higher rates of breastfeeding initiation, exclusivity, and duration, as well as improved skin-to-skin contact, mother–infant bonding, and maternal breastfeeding confidence [8,9,10,11,12]. BFHI-aligned practices also support early initiation of breastfeeding within the first hour of birth, contributing to improved neonatal physiological stability and breastfeeding outcomes [13,14]. In addition, BFHI strengthens breastfeeding education and supports parents in recognizing feeding cues and accessing timely breastfeeding assistance [15].

Despite the adoption of BFHI in more than 150 countries, its implementation remains inconsistent in Canada, with relatively few BFHI-designated facilities and substantial interprovincial variation in breastfeeding policies, practices, and support services [16,17]. Previous Canadian studies have examined breastfeeding promotion, selected BFHI practices, and challenges to breastfeeding support within individual hospitals or provinces [14,16,17]. Collectively, these studies suggest that BFHI-aligned practices vary across healthcare settings and are influenced by organizational, workforce, and contextual factors [14,16,17]. However, limited qualitative research has examined how these factors interact across multiple levels of the health system to influence BFHI implementation and scale-up, particularly from the perspectives of diverse stakeholders, including mothers, healthcare professionals, and health system stakeholders.

The gap is particularly relevant in Saskatchewan, where only one BFHI-designated facility exists among approximately 70 healthcare settings [18]. The limited uptake is concerning given provincial breastfeeding patterns, where although 94.9% of mothers initiate breastfeeding, many discontinue within the first six months, and only 38.8% exclusively breastfeed for the recommended duration [19]. Previous qualitative research in Saskatchewan has identified inconsistencies in breastfeeding support within healthcare settings, including variation in counselling practices, healthcare provider support, and early introduction of formula feeding [20,21,22]. While these studies provide important insights into breastfeeding experiences and service delivery, they have not specifically examined the broader health system factors influencing BFHI implementation and scale-up or integrated perspectives across multiple stakeholder groups.

Accordingly, there remains a need to better understand the barriers and facilitators influencing the implementation and scale-up of BFHI within Saskatchewan healthcare settings. Previous research on the implementation of breastfeeding-supportive practices suggests that organizational capacity, leadership and governance, workforce readiness, consistency of clinical practices, continuity of care, and equitable access to breastfeeding support may be relevant to implementation [14,16,17,20,21,22]. However, there is limited understanding of the barriers and facilitators influencing BFHI implementation and scale-up across stakeholder groups and levels of the Saskatchewan healthcare system. Examining these perspectives across multiple stakeholder groups can provide a more comprehensive understanding of the context in which BFHI implementation and scale-up occur and inform efforts to strengthen breastfeeding-supportive maternity and newborn care. Therefore, this study aimed to explore the barriers and facilitators influencing the implementation and scale-up of the BFHI in Saskatchewan, Canada.

## 2. Materials and Methods

### 2.1. Study Design

This study employed a qualitative descriptive design to explore the barriers and facilitators to the implementation and scale-up of the Baby-Friendly Hospital Initiative (BFHI) in healthcare settings in Saskatchewan, Canada. A qualitative descriptive approach was selected to generate a comprehensive and contextually grounded understanding of health system, provider, and maternal experiences related to BFHI implementation [23]. The study was guided by Bronfenbrenner’s socioecological model [24], which conceptualizes health behaviours and system interactions as occurring across interrelated environmental levels, including individual, interpersonal, organizational, community, and policy contexts. This framework was used to inform data collection and analysis by shaping the interview guide and guiding the organization of codes and emerging themes. It enabled examination of how breastfeeding-related practices are influenced by interactions between mothers and healthcare systems, as well as broader structural and policy conditions. This multi-level perspective provided a structured lens for identifying how facilitators and barriers to BFHI implementation operate across different levels of the health system in Saskatchewan. The reporting of this qualitative study follows the Consolidated Criteria for Reporting Qualitative Research (COREQ) guidelines.

### 2.2. Study Setting

The study was conducted in Saskatchewan, Canada, within healthcare settings providing maternity, postpartum, neonatal, and community-based maternal-child health services. The province includes a mix of urban, rural, and remote health regions, providing a diverse contextual environment for examining BFHI implementation.

### 2.3. Ethical Considerations

Ethical approval for the study was obtained from the University of Regina’s Ethics Review Board [REB # 2025-1260]. All participants provided informed consent prior to participation. Confidentiality was maintained by removing identifying information from transcripts and storing data securely in password-protected systems accessible only to the research team.

### 2.4. Participant Recruitment and Sampling

Altogether, the sample comprised 26 participants, including breastfeeding mothers and multidisciplinary healthcare providers (e.g., nurses, nutritionists, lactation consultants, and public health professionals) involved in maternal and newborn care. Participants represented both urban and rural Saskatchewan communities, including major urban centres and smaller communities, and represented three intersecting perspectives, i.e., mothers with lived breastfeeding experience, healthcare providers supporting infant feeding, and individuals occupying both roles.

A combination of emergent sampling and maximum variation sampling was used to ensure both depth and diversity of perspectives. Emergent sampling allowed for iterative recruitment of information-rich participants actively engaged in breastfeeding care, while maximum variation sampling ensured inclusion of participants across different professional roles, geographic regions, and socio-demographic backgrounds.

Eligible participants were adults (≥18 years of age) who resided or worked in Saskatchewan (Canada) and either had lived experience of breastfeeding within the provincial healthcare system or professional experience providing maternal, newborn, or breastfeeding care. Individuals who were unable to communicate in English or who did not meet these eligibility criteria were excluded.

Participant recruitment was facilitated through collaboration with the provincial health authority, professional networks, knowledge users, and patient partners within Saskatchewan’s healthcare system. Study invitations describing the purpose of the study, eligibility criteria, and participation requirements were disseminated through email communication, notice boards, and social media of these networks to potentially eligible participants. Participation was voluntary, and individuals who did not wish to participate were not required to contact the research team. Individuals who were interested in participating contacted the Principal Investigator (PI) directly, after which eligibility was confirmed and informed consent procedures were completed prior to participation. Recruitment proceeded concurrently with data analysis and continued until thematic saturation was achieved.

Saturation was assessed through ongoing review of interview transcripts and coding following successive interviews. The PI and trained Research Assistant (RA) met regularly to compare emerging codes and themes, and recruitment ceased when additional interviews yielded no new codes, concepts, or thematic insights across the participant groups, and consensus was reached that sufficient informational depth had been achieved to address the study objectives.

The PI is a female Professor of Nursing with expertise in maternal and child health, breastfeeding research, and implementation of the Baby-Friendly Hospital Initiative (BFHI). The PI has extensive experience conducting qualitative health research and has worked collaboratively with healthcare organizations and communities to improve breastfeeding policies, practices, and services. The PI was professionally acquainted with some healthcare provider participants through previous collaborations, provincial breastfeeding initiatives, and professional networks. However, no supervisory, evaluative, or dependent relationships existed between the PI and participants, and the PI had no prior relationship with participating mothers before recruitment. Participants were informed of the PI’s professional role, research interests, and the purpose of the study before providing informed consent.

To promote reflexivity and minimize potential researcher influence, the PI maintained reflective journals throughout data collection and analysis to document assumptions, methodological decisions, and emerging interpretations. Regular discussions with the trained RA, knowledge users, and patient partners provided opportunities to critically examine interpretations, challenge assumptions, and ensure that findings remained grounded in participants’ experiences.

### 2.5. Data Collection

Data were collected through semi-structured in-depth interviews conducted during 2025. An interview guide was developed based on the study objectives and guided by Bronfenbrenner’s socioecological model [24] to explore multi-level influences shaping BFHI implementation within Saskatchewan healthcare settings. The guide included open-ended questions examining participants’ experiences, perceptions, facilitators, barriers, and recommendations related to breastfeeding support and BFHI implementation. The interview process began with broad exploratory questions, including: “Tell us about your experiences and perceptions regarding breastfeeding care and services within Saskatchewan healthcare settings.” Follow-up probes explored organizational practices, healthcare provider support, access to breastfeeding resources, continuity of care, clinical practices, and system-level factors influencing the delivery of Baby-Friendly care.

Interviews were conducted by the PI and a trained RA either in person or virtually, depending on participant preference and feasibility. Both in-person and virtual interviews followed the same semi-structured interview guide and included identical core questions and follow-up probes to ensure consistency across interviews. Interviews lasted approximately 40–60 min, allowing sufficient time for participants to describe their experiences, perspectives, and recommendations related to BFHI implementation. All interviews were conducted in English, audio-recorded with participant consent, and professionally transcribed. The research team maintained reflective journals to capture contextual observations, reflexive insights, and emerging analytical considerations throughout the data collection process. Following each interview, the PI and RA reviewed field notes and the interview transcript to identify emerging concepts and assess whether additional interviews were contributing new insights. Regular analytic meetings were held throughout data collection to review coding progression and determine whether thematic saturation had been reached.

### 2.6. Data Analysis

Data analysis was conducted iteratively throughout the study, beginning concurrently with data collection. Initial coding and analytic discussions informed ongoing recruitment and assessment of thematic saturation. Recruitment concluded when successive interviews generated no new codes or conceptual insights and the research team reached consensus that the data adequately addressed the study objectives. The PI and RA conducted the analysis manually using Braun and Clarke’s six-phase thematic analysis [25]. All interview transcripts, field notes, and reflective journals were reviewed repeatedly to achieve familiarity with the data while documenting initial observations and emerging patterns related to barriers and facilitators influencing Baby-Friendly Hospital Initiative (BFHI) implementation.

The analysis followed six interconnected phases. First, the PI and RA independently reviewed the data and generated initial codes that captured meaningful concepts relevant to participants’ experiences. Second, codes were compared, discussed, and refined through an iterative process of constant comparison to ensure consistency and depth of interpretation. Third, related codes were organized into preliminary categories and potential themes representing broader patterns across participant groups and healthcare settings. Fourth, these themes were reviewed against the coded extracts and the entire dataset to ensure coherence, internal consistency, and distinction between themes. Fifth, themes were refined, defined, and named to accurately reflect the underlying meaning of the data. Finally, the themes were interpreted through collaborative discussions involving the PI, RA, knowledge users, and patient partners to ensure they comprehensively represented participants’ experiences of BFHI implementation within Saskatchewan’s healthcare system.

The PI and RA met regularly to compare interpretations, discuss discrepancies, refine code definitions, and revise the coding framework as needed. Disagreements regarding coding decisions or theme development were resolved through discussion and consensus between the PI and RA, with additional input from knowledge users and patient partners when interpretation required broader contextual perspectives. Consensus meetings occurred throughout the analytic process rather than only after completion of initial coding, allowing continuous refinement of codes, categories, and themes. Table 1 provides an illustrative example of the analytic progression from initial codes to final themes.

### 2.7. Rigor and Trustworthiness

The trustworthiness of the study was established using Lincoln and Guba’s criteria [26] of credibility, dependability, confirmability, and transferability. Credibility was enhanced through triangulation of participant groups representing diverse perspectives, including mothers with breastfeeding experience, healthcare providers, lactation specialists, and health system stakeholders. Credibility was further supported through prolonged engagement with the data, iterative review of transcripts, member checking with participants, reflexive journaling and ongoing discussions among the research team during the analytic process to refine interpretations and ensure alignment with participants’ experiences. Following each interview, participants were provided with a copy of their interview transcript and invited to review the content for accuracy, completeness, and clarification. Participants were given the opportunity to provide corrections, additional context, or feedback regarding their accounts, and any suggested revisions or clarifications were incorporated into the final dataset prior to analysis. This process strengthened the authenticity of the findings by ensuring that participants’ perspectives were accurately represented.

Dependability was strengthened through a systematic and transparent analytic approach, including structured coding procedures, documentation of analytic decisions, and maintenance of an audit trail throughout data collection and analysis. Regular analytic meetings between the PI and RA supported consistency in coding decisions, while discussions with knowledge users and patient partners contributed to contextual interpretation of the findings.

Confirmability was promoted through researcher reflexivity, reflexive journaling, and collaborative interpretation of findings among multiple researchers and stakeholders to acknowledge and address potential researcher biases. Given that the PI conducted participant interviews and was involved in the analytic process, reflexivity was incorporated throughout the study to acknowledge and manage potential researcher influence. The PI maintained reflective journals documenting personal reflections, assumptions, methodological decisions, and emerging interpretations throughout data collection and analysis. Regular discussions between the PI, RA, knowledge users, and patient partners provided opportunities to critically examine interpretations, challenge assumptions, and consider alternative explanations of the findings. This collaborative approach supported reflexive awareness and helped ensure that themes remained grounded in participants’ accounts rather than researchers’ preconceptions.

Transferability was supported through the provision of rich, thick descriptions of participants’ characteristics, experiences, and the Saskatchewan healthcare context, allowing readers to assess the relevance and applicability of findings to other settings.

## 3. Results

### 3.1. Demographic Characteristics of Participants

Table 2 summarizes characteristics of the 26 participants, who represented diverse maternal and professional perspectives on breastfeeding support across Saskatchewan. Participants comprised mothers (*n* = 11, 42.3%), mothers who were also healthcare professionals (*n* = 6, 23.1%), and healthcare professionals without maternal experience (*n* = 9, 34.6%) working in maternal-child settings across Saskatchewan. Overall, 17 participants (65.4%) were mothers, while 15 (57.7%) were healthcare professionals.

Among the 26 participants, 15 healthcare professionals represented diverse roles and practice settings across the maternal, neonatal, acute care, public health, nutrition, and community sectors. Their professional roles included registered nurses or licensed practical nurses (*n* = 9), registered nurses or licensed practical nurses with International Board-Certified Lactation Consultant (IBCLC) certification (*n* = 4), and pediatric dietitians or public health nutritionists (*n* = 2). Their practice settings included public health and community visiting programs (*n* = 4), hospital-based maternal care, including prenatal, labor and birth, and postnatal units (*n* = 3), acute care hospital settings (*n* = 3), neonatal intensive care units (NICUs) (*n* = 2), nutrition services across hospital and community settings (*n* = 2), and private lactation practice in the community (*n* = 1).

Participants represented a range of urban, regional, and smaller communities across Saskatchewan, including Moose Jaw (*n* = 8, 30.8%), Regina (*n* = 6, 23.1%), Saskatoon (*n* = 5, 19.2%), Prince Albert (*n* = 2, 7.7%), and smaller communities (*n* = 5, 19.2%), including La Ronge, Yorkton, White City, Balgonie, and Chaplin/Central Butte.

Among the 17 mothers, eight reported no clinical concerns related to their infants, while the remaining mothers reported infant clinical concerns, including tongue-tie/lip-tie (*n* = 4), jaundice (*n* = 3), or NICU admission (*n* = 2).

Together, the combination of maternal lived experiences, clinical expertise and knowledge, and perspectives from diverse Saskatchewan communities provided a broad understanding of factors influencing breastfeeding experiences and support across the province.

### 3.2. Barriers and Facilitators to the Implementation and Scale-Up of the BFHI

Interviews with mothers and healthcare professionals identified five interconnected themes influencing BFHI implementation and scale-up in Saskatchewan. Table 3 summarizes the key facilitators and barriers identified within each theme, their corresponding socioecological levels, and the participant perspectives represented across the findings.

#### 3.2.1. Theme 1: Leadership and Governance as Foundations for BFHI Implementation

Participants emphasized that leadership commitment, organizational structures, and governance mechanisms were central to embedding BFHI principles into clinical practice. However, sustainability was challenged when leadership support was inconsistent or dependent on individual champions.

##### Leadership Engagement and Organizational Commitment

Supportive leadership was described as an important facilitator of BFHI implementation. Participants highlighted that managers and organizational leaders who prioritized breastfeeding created an environment where staff felt encouraged to integrate BFHI-aligned practices into routine care. A healthcare professional who was also a mother shared, “We’re striving to be a Baby-Friendly Hospital. It’s very challenging to get that status. We’re striving to be… I have a really, really amazing manager that is very supportive” (Participant 5).

Participants also described institutional recognition initiatives as reinforcing organizational commitment to breastfeeding. One healthcare professional explained, “I want to say April or May of 2025, that we received our gold status for the breastfeeding recognition program, which was very exciting” (Participant 21).

These initiatives were perceived as providing motivation and strengthening institutional visibility of breastfeeding support.

##### Governance Structures and Standardized Approaches

Participants identified provincial guidelines, breastfeeding committees, and organizational policies as important mechanisms for supporting consistency in BFHI implementation. Provincial breastfeeding guidelines were viewed as valuable resources for reducing practice variation and guiding clinical decision-making.

A healthcare professional with IBCLC certification explained, “We follow the provincial breastfeeding guidelines that were released, I believe in January 2024. We do follow the provincial breastfeeding guidelines as well as the algorithm that came through, which is very helpful, that kind of goes into more detail regarding medical indications for supplementation and so forth” (Participant 20).

Interdisciplinary governance structures were also described as facilitating collaboration across healthcare services. One healthcare professional who was also a mother highlighted the role of breastfeeding committees in advocating for need-based breastfeeding support: “We have a breastfeeding committee in our community… they kind of look at how we can all discuss, like a roundtable [discussion] with our women’s health unit, our pediatrics unit, and our public health” (Participant 26).

##### Leadership Turnover and Challenges to Sustainability

Although leadership was recognized as an important facilitator, participants described concerns regarding sustainability when key individuals left their positions or institutional priorities changed. Changes in leadership were perceived to affect the continuity of BFHI efforts and organizational knowledge.

A healthcare professional with IBCLC certification stated, “We had a manager who was very BFI-friendly, but after that our managers changed so much that they probably don’t even understand what the BFI is” (Participant 11).

Participants emphasized the need for sustained organizational commitment beyond individual leaders to maintain breastfeeding initiatives over time.

Overall, leadership engagement, governance structures, and provincial alignment were seen as critical but fragile enablers of BFHI scale-up. These findings suggest that BFHI scale-up is not solely dependent on guidelines or policy availability, but on sustained leadership capacity and institutional memory. Without stable governance, implementation risks becoming fragmented and person-dependent rather than system-embedded.

#### 3.2.2. Theme 2: Workforce Capacity, Competency and Practice Consistency as Determinants of Care Delivery

Participants described healthcare providers as central to BFHI implementation, with breastfeeding support influenced by staff availability, clinical expertise, and consistency of practice. Although many participants recognized strong professional commitment to breastfeeding, workforce limitations contributed to variation in care experiences.

##### Skilled and Responsive Breastfeeding Support

Mothers frequently identified knowledgeable and supportive healthcare providers as important facilitators of breastfeeding initiation. Hands-on assistance, including support with positioning, latch, and feeding confidence, was described as particularly valuable during the early postpartum period.

One mother who was also a healthcare professional and had babies with a history of jaundice and tongue tie shared, “They [nurses] said, ‘Call anytime. Let me know when you’re feeding; I’ll come take a look.’ They would place baby to my breast, help me get the latch down, and try different holds with me. I did find the nurses very knowledgeable” (Participant 18).

Participants described individualized support as helping mothers feel more confident and capable during breastfeeding initiation.

##### Staffing Pressures and Inconsistent Breastfeeding Practices

Despite recognizing the importance of breastfeeding support, participants described staffing shortages and competing clinical responsibilities as barriers to consistent BFHI implementation. Healthcare providers explained that workload pressures sometimes limited their ability to provide adequate breastfeeding assistance.

A healthcare professional shared, “The demands of your day-to-day care take away from supporting the initiation and support that parents need with breastfeeding” (Participant 14).

Participants also described variation in breastfeeding support depending on the provider, shift, or clinical context. A healthcare professional stated, “There’s a huge discrepancy between what happens on days versus what happens on nights, and this goes back to not having enough hands and being short-staffed” (Participant 21).

Findings suggest that inconsistent practices created uncertainty for families and affected the continuity of breastfeeding guidance.

##### Variation in Professional Recommendations and Lactation Expertise

Participants identified differences in recommendations among healthcare professionals as another challenge to consistent BFHI implementation. Some described situations where supplementation recommendations differed from mothers’ breastfeeding intentions.

Participants described how hierarchical decision-making structures affected feeding trajectories. A healthcare provider explained, “I have one physician that goes up to parents and says, ‘I know you want to exclusively breastfeed, but are you okay with a little bit of formula?’” (Participant 5).

This variability contributed to confusion for families and weakened the continuity of breastfeeding support during a critical physiological and emotional period. Such instances reinforced inconsistent messaging across professional groups, placing parents in situations where breastfeeding decisions were shaped by professional variation rather than unified clinical guidance.

In addition to staffing and hierarchical variation, systemic constraints in workforce development were identified as limiting the expansion and sustainability of breastfeeding expertise. Participants highlighted financial barriers to professional certification and advancement as a structural impediment to strengthening lactation support capacity. One healthcare professional shared, “The biggest barrier is that IBCLC certification is not paid, and those in that role are not paid as lactation consultants” (Participant 21).

#### 3.2.3. Theme 3: Continuity of Breastfeeding Support Across Care Settings

Participants emphasized that breastfeeding support extended beyond the immediate postpartum hospitalization and depended on effective transitions between hospital, public health, and community-based services. Continuity of care was identified as an important facilitator of sustained breastfeeding; however, gaps in follow-up and service coordination limited consistent support for some families.

##### Public Health and Community-Based Supports as Facilitators of Breastfeeding Continuation

Participants described public health nursing services, home visiting programs, and community-based breastfeeding supports as valuable resources after hospital discharge. These services provided mothers with ongoing clinical guidance, reassurance, and connections to additional breastfeeding resources.

A mother shared, “Public health nurses come and check on you after [your hospital discharge] through the maternal visiting program… they’re the ones that have led me to lactation every time” (Participant 18).

Home visits were particularly valued because they allowed breastfeeding support to occur within the mother’s own environment. Mothers described these visits as providing emotional reassurance and individualized assistance.

One mother explained, “The home visit is a valuable thing. I took advantage of it. They’re nice and patient and slow in those visits. They want to connect and help in all ways” (Participant 8).

Participants also highlighted peer-based supports, such as breastfeeding groups and community programs, as opportunities for mothers to receive encouragement and normalize breastfeeding experiences. A healthcare professional described, “It’s a casual time of connection over coffee for moms to connect with other moms” (Participant 6).

Access to practical resources and tangible supports, such as access to breast pumps through public health services, was also identified as facilitating breastfeeding continuation. One mother shared, “She [healthcare provider] arranged me a hospital-grade breast pump in the home for free. That was a good experience” (Participant 23).

##### Gaps in Follow-Up Care and Referral Pathways

Although participants recognized the importance of community-based services, they described limitations in the availability and intensity of follow-up support. Some mothers reported requiring more frequent assistance than services were able to provide.

A mother who was also a healthcare professional stated, “Public health nurses do come and see mothers initially, but they’re not always able to go as frequently as what some people would need” (Participant 25).

Healthcare professionals also described challenges related to referral pathways and coordination between healthcare sectors. While referral systems existed, participants noted variability in how effectively they supported mothers with ongoing breastfeeding challenges. Participants emphasized that strengthening connections between hospital and community services was important for ensuring mothers received timely breastfeeding support after discharge.

#### 3.2.4. Theme 4: Breastfeeding as a Psychosocially Embedded Maternal Experience

Participants highlighted that breastfeeding experiences were shaped by maternal emotional wellbeing, respectful care, access to information, cultural expectations, and broader social circumstances. Breastfeeding support was viewed as extending beyond clinical skills to include recognition of mothers’ lived experiences and circumstances.

##### Maternal Mental Health, Emotional Vulnerability, and Respectful Care

Participants identified maternal mental health as an important factor influencing breastfeeding confidence and continuation. Healthcare providers described the postpartum period as a time of increased emotional vulnerability, where psychological wellbeing influenced breastfeeding experiences.

A healthcare provider shared, “We see mental health playing a huge role postnatally, and it definitely affects the supply of breast milk and the motivation to continue” (Participant 12).

Mothers emphasized the importance of compassionate and respectful approaches during this vulnerable period. One mother stated, “You’re just so fragile postpartum. Maybe there could have been a more gentle approach” (Participant 10).

Respectful communication and support for maternal preferences were described as important facilitators. A mother shared, “From the beginning they asked if breastfeeding was my plan, and everyone seemed pretty supportive of that” (Participant 9).

##### Breastfeeding Education, Preparation, and Early Postpartum Transitions

Participants described challenges related to limited breastfeeding education during short hospital stays. Some mothers reported receiving insufficient information or feeling overwhelmed by the amount of information provided immediately after childbirth.

Early discharge practices (within 24 hours of childbirth) were identified as creating challenges for mothers who required additional assistance establishing breastfeeding. A mother explained, “It’s a lot when you just gave birth, and you’re getting ready to go home, and then they have a lot of paperwork for you to go through” (Participant 9).

Some participants reported insufficient breastfeeding education during their short-term hospital stay for childbirth. While recalling her hospital discharge time after childbirth, a mother shared, “When we were going home, a nurse did come in and went over some discharge papers, but I don’t think anything was said about breastfeeding” (Participant 19).

##### Cultural, Social, and Workplace Influences on Breastfeeding Continuation

Participants described breastfeeding as being influenced by cultural expectations, family support systems, and social circumstances. Mothers from migrant backgrounds highlighted differences between their previous experiences and the support available in Canada.

An immigrant mother shared, “In our home country, I can choose the doctor, or the gender of the doctor. But here I didn’t get that choice… it was like a cultural shock. In our culture, the moms, the grandmoms, and even the nurses and doctors are very helpful. They guide you, and our moms and grandmoms help you with breastfeeding and everything. But here I am alone” (Participant 15).

Return-to-work expectations were also identified as barriers to sustaining breastfeeding. A healthcare provider explained, “They [mothers] value breastfeeding a lot, but there are challenges in continuing with that, especially when they have to come back to work really soon” (Participant 6).

These findings highlight that breastfeeding decisions and experiences were influenced by social conditions beyond healthcare encounters.

#### 3.2.5. Theme 5: Structural and Societal Factors Shaping BFHI Scale-Up

Participants identified broader structural conditions that influenced BFHI implementation, including healthcare environments, geographic accessibility, societal norms, sociocultural normalization of formula feeding, and system-level accountability. These factors shaped both the capacity of healthcare services to provide breastfeeding support and mothers’ ability to access and continue breastfeeding.

##### Physical Environments and Geographic Inequities

Participants described hospital environments that sometimes conflicted with BFHI principles, particularly in relation to rooming-in and privacy. Healthcare providers noted that physical infrastructure (mainly crowded spaces and reduced privacy) could limit opportunities for breastfeeding support.

A healthcare professional explained, “If you have mom in her bed, all the newborns in their cots, and then all the support people, it’s very squishy and very loud. The physical layout of our hospital takes away from our rooming in, which is ultimately where we want to be” (Participant 20).

Geographic barriers were particularly emphasized for rural and remote families. Limited transportation options and distance from services affected access to breastfeeding support.

##### Formula Normalization and Competing Infant Feeding Messages

Participants discussed societal influences that shaped perceptions of infant feeding. Healthcare providers described formula marketing through its widespread availability and provision of free samples/coupons as contributing factors to the normalization of formula feeding.

One healthcare provider stated, “Formula companies do a very good job of making sure people know it’s a great way to feed your baby. Some doctors’ offices have formula samples that they’re free to hand out” (Participant 7).

Some mothers described situations where formula was introduced without sufficient discussion or consideration of their breastfeeding goals.

A mother who gave birth to her baby at 35 weeks shared, “She [speech-language pathologist] just came in with a bottle of formula, didn’t ask any questions, and started feeding him the bottle, which was bizarre to me. My goal was to breastfeed him [my baby]” (Participant 17).

Participants emphasized the importance of shared decision-making and ensuring that supplementation decisions align with maternal preferences and clinical indications.

##### Policy Gaps, Surveillance Limitations, and Fragmented Systems

Participants identified gaps in provincial breastfeeding surveillance and accountability as barriers to coordinated improvement. Lack of consistent data collection was described as limiting the ability to monitor breastfeeding outcomes and guide quality improvement initiatives.

A healthcare professional stated, “A lot of initiatives are not data-driven. We don’t have universal breastfeeding statistics across the province” (Participant 14).

Participants also highlighted challenges related to access to lactation services and timely referrals. Some mothers described delays in receiving specialized breastfeeding assistance that contributed to unresolved breastfeeding challenges. A mother shared, “We don’t have a lot of options for when we’re needing to refer for lactation consulting” (Participant 12).

Across themes, participants described BFHI implementation as being influenced by multiple interacting factors, including leadership commitment, workforce capacity, continuity of care, maternal experiences, and broader structural conditions. Facilitators such as supportive leadership, skilled providers, and community-based services strengthened breastfeeding support, whereas staffing pressures, inconsistent practices, fragmented services, and structural inequities limited consistent implementation.

## 4. Discussion

This study provides a comprehensive analysis of the barriers and facilitators influencing the implementation and scale-up of the Baby-Friendly Hospital Initiative (BFHI) in Saskatchewan, Canada. While previous studies have largely examined individual barriers to breastfeeding support or evaluated specific BFHI practices, this study demonstrates that successful implementation of BFHI is shaped by dynamic interactions across organizational, workforce, community, and broader health system contexts. These findings are consistent with the global literature [27,28,29]. The findings illustrate that implementation is neither linear nor solely dependent on individual healthcare providers. Instead, BFHI functions as a complex health systems intervention in which leadership, governance, workforce capacity, continuity of care, maternal psychosocial experiences, and structural conditions collectively determine whether evidence-based breastfeeding practices become embedded in routine maternity care.

Bronfenbrenner’s socioecological model [24] provided a useful lens for understanding how barriers and facilitators to BFHI implementation and scale-up were situated across interconnected individual, interpersonal, organizational, community, and health system contexts in Saskatchewan. Consistent with Bronfenbrenner’s socioecological model [24], the findings emphasize that health behaviors and healthcare practices are influenced by multiple interacting levels of the environment, ranging from individual experiences to organizational and policy contexts. They also reinforce the WHO and UNICEF position that successful implementation of the Ten Steps to Successful Breastfeeding requires coordinated action across the entire health system rather than isolated clinical interventions [6]. Across interviews, participants described a growing movement toward Baby-Friendly care in Saskatchewan that is already visible in practice, even though relatively few facilities have a formal BFHI designation [30].

### 4.1. Leadership and Governance as Foundations for BFHI Implementation (Theme 1)

The findings identify leadership and governance as fundamental determinants of BFHI implementation and scale-up. Participants consistently described supportive managers, provincial breastfeeding policies, interdisciplinary committees, and institutional recognition programs as creating the organizational conditions necessary for implementing Baby-Friendly practices. These findings reinforce the central importance of organizational leadership within implementation science, where leadership engagement is recognized as a key determinant of the successful adoption, implementation, and sustainability of complex healthcare interventions [31,32].

These findings align with Step 1, highlighting the importance of organizational systems that translate breastfeeding policy into routine practice [6]. Participants viewed Saskatchewan’s provincial breastfeeding guidelines as providing an evidence-based framework that legitimized breastfeeding practices across healthcare settings. Similarly, breastfeeding advisory committees facilitated collaboration across maternity services, pediatrics, neonatal care, and public health, illustrating how governance structures can translate policy into coordinated clinical practice. Despite these important facilitators, participants consistently described leadership support as fragile and highly dependent on individual champions. Leadership turnover frequently resulted in declining organizational commitment, reduced visibility of breastfeeding initiatives, and loss of institutional knowledge surrounding BFHI implementation. Similar challenges have been reported internationally, where sustaining BFHI implementation requires long-term organizational commitment, stable governance, and ongoing leadership engagement beyond the efforts of individual champions [6,33]. This highlights the importance of institutionalizing BFHI within health system governance through sustained leadership development, organizational accountability, and continuous quality improvement mechanisms to ensure implementation remains resilient despite administrative change.

### 4.2. Workforce Capacity, Competency, and Practice Consistency as Determinants of Care Delivery (Theme 2)

While leadership and governance established the organizational foundation for BFHI implementation, workforce capacity emerged as an important determinant of whether Baby-Friendly principles were translated into consistent clinical practice. Participants emphasized that breastfeeding support depended not only on the presence of breastfeeding policies or guidelines but on the availability of knowledgeable, skilled, and accessible healthcare providers who could provide individualized assistance during the critical early postpartum period. Mothers particularly valued hands-on support from nurses, lactation consultants, midwives, and neonatal staff, describing assistance with positioning, latch assessment, milk expression, and problem-solving as essential for developing breastfeeding confidence. These findings align with systematic reviews demonstrating that skilled healthcare professional support improves breastfeeding initiation, exclusivity, and continuation [28,34,35].

The importance of workforce capacity was reflected across multiple components of the WHO/UNICEF Ten Steps to Successful Breastfeeding. Step 2 emphasizes ensuring that healthcare staff possess sufficient knowledge, competence, and skills to implement breastfeeding-supportive practices [6]. Thus, workforce readiness represents a foundational requirement through which multiple BFHI practices are operationalized. Although participants described strong professional commitment to breastfeeding support, they also emphasized that knowledge alone was insufficient without opportunities for experiential learning, mentorship, and ongoing skill development.

Despite this commitment, BFHI implementation was characterized by substantial variability across providers, shifts, and healthcare settings. Participants described differences in breastfeeding counselling, supplementation decisions, and clinical recommendations that resulted in inconsistent experiences for families. Such variation was particularly evident when mothers encountered conflicting messages from different healthcare professionals, creating uncertainty during a vulnerable period. Similar challenges have been reported internationally, where inconsistent provider practices and variable interpretation of breastfeeding recommendations have weakened implementation fidelity and reduced parental confidence [33,36].

The variation in supplementation decisions has implications for consistent application of evidence-based supplementation criteria [6]. Participants described situations in which supplementation decisions were influenced not only by clinical indications but also by provider preferences, workload pressures, and differing professional interpretations of breastfeeding challenges. Consequently, supplementation could become a response to system constraints rather than a carefully considered clinical decision. This finding suggests that successful implementation of Step 6 requires shared interdisciplinary understanding, standardized supplementation protocols, and consistent application of evidence-based criteria across professional groups.

Importantly, participants did not attribute variation in practice to a lack of commitment among healthcare providers. Rather, they identified structural workforce constraints that limited the consistent delivery of BFHI-aligned care. Staffing shortages, competing clinical priorities, limited availability of lactation consultants, and high patient workloads reduced opportunities for providers to observe feeds, provide responsive counselling, support early breastfeeding initiation, and address challenges before supplementation was introduced. Under these conditions, breastfeeding support became contingent on who was available, when support was requested, and the clinical pressures present at the time, rather than being reliably embedded within routine maternity care. The findings further highlight that sustainable BFHI implementation requires investment in workforce development as a system-level priority. Participants identified barriers to expanding breastfeeding expertise, including limited mentorship opportunities, challenges maintaining clinical competency, and financial barriers to obtaining IBCLC certification. Similar concerns have been identified internationally, where insufficient investment in breastfeeding-specific workforce development has constrained long-term BFHI sustainability despite widespread recognition of its benefits [28,31,33,37,38,39,40,41,42]. Addressing these challenges requires strategies beyond continuing education alone, including protected training time, funded certification pathways, mentorship models, and staffing structures that recognize breastfeeding support as an essential component of maternity care. From a health systems perspective, breastfeeding support should therefore be conceptualized not as an additional responsibility assigned to individual providers, but as an essential healthcare service requiring sustained investment in staffing, competency development, interdisciplinary collaboration, and standardized clinical practice.

### 4.3. Continuity of Care as an Essential but Vulnerable System Pathway (Theme 3)

A central contribution of this study is the identification of continuity of care as a critical, yet structurally fragile, bridge connecting hospital-based breastfeeding support with sustained breastfeeding following discharge. Participants consistently emphasized that breastfeeding outcomes were influenced not only by the quality of care received during childbirth but also by the availability of coordinated post-discharge support through public health nursing, home-visiting programs, lactation consultants, and community-based peer networks. These findings reinforce growing evidence that breastfeeding should be understood as a continuum of care extending beyond maternity services rather than a discrete hospital-based intervention [34,43].

This finding is consistent with Step 10, which emphasizes coordinated discharge planning and timely community referral [6]. Participants described home-visiting programs, public health nurses, Baby Cafés, and breastfeeding peer groups as valuable extensions of hospital care that provided practical guidance, emotional reassurance, and early identification of breastfeeding challenges. These community-based services helped mothers navigate common difficulties during the transition from hospital to home and reinforced breastfeeding confidence during a period of heightened vulnerability. Public health follow-up created opportunities to develop trusting relationships, assess maternal wellbeing, address breastfeeding concerns within the home environment, and facilitate referrals to specialized services when needed. This relational dimension of continuity is increasingly recognized within maternal-child healthcare, where sustained provider relationships promote maternal confidence, responsiveness to emerging needs, and greater engagement with health services [35,44,45,46]. The findings therefore suggest that continuity of care should be conceptualized not only as service coordination but also as an ongoing therapeutic relationship that supports breastfeeding through changing maternal and infant needs. Despite these important strengths, continuity of care remained structurally vulnerable. Participants consistently described limitations in workforce capacity, variable availability of public health follow-up, and inconsistent access to lactation consultants across regions. Although referral systems existed, their effectiveness depended heavily on local staffing resources, service availability, and geographical accessibility. Consequently, mothers frequently experienced delays in accessing specialized breastfeeding support, particularly when challenges emerged after discharge. These findings echo previous Canadian and international research demonstrating that fragmentation between hospital and community services remains a significant barrier to sustained breastfeeding support [33,43]. Public health services often addressed breastfeeding difficulties that had not been fully resolved during the hospital stay, including problems with latch, milk supply, supplementation, and maternal confidence. For successful BFHI scale-up, continuity of care must therefore be strengthened through greater integration between maternity services, neonatal care, primary care, public health, and community organizations. These findings suggest that coordinated referral pathways, shared communication systems, expanded community lactation services, and equitable access to post-discharge support, particularly in rural and remote communities, warrant consideration in future BFHI implementation planning and evaluation.

### 4.4. Breastfeeding as a Psychosocially Embedded Maternal Experience (Theme 4)

A key finding of this study is that breastfeeding cannot be understood solely as a clinical behaviour shaped by healthcare interventions. Rather, it emerged as a psychosocially embedded practice influenced by emotional wellbeing, family circumstances, cultural expectations, employment demands, and broader social realities. These findings extend traditional interpretations of BFHI implementation by illustrating that successful implementation depends not only on adherence to clinical guidelines but also on the ability of health systems to respond to the complex lived experiences of mothers during the postpartum period. Participants’ accounts suggest that these principles are only partially realized in Saskatchewan because psychosocial support remains constrained by early discharge, limited follow-up capacity, and competing health system pressures.

Maternal mental health emerged as one of the strongest cross-cutting influences on breastfeeding initiation and continuation. Participants described how postpartum anxiety, emotional exhaustion, stress, and depression affected maternal confidence, breastfeeding motivation, and perceived milk supply. These findings are consistent with previous research demonstrating a bidirectional relationship between breastfeeding and maternal mental health, whereby psychological distress may contribute to breastfeeding difficulties while unsuccessful breastfeeding experiences may further exacerbate emotional distress [47,48,49,50]. Rather than viewing breastfeeding support and maternal mental health as separate domains of care, the findings suggest that they should be integrated within comprehensive postpartum services. Participants consistently valued healthcare providers who demonstrated patience, empathy, and encouragement, suggesting that relational aspects of care were as important as clinical expertise in facilitating breastfeeding success. These findings reinforce the importance of responsive and person-centred breastfeeding support [34,51]. Participants further emphasized the importance of individualized care that respected maternal circumstances, acknowledged breastfeeding challenges, and supported mothers in making informed decisions based on their own needs and those of their infants. This individualized approach contrasts with protocol-driven care and highlights the importance of clinical flexibility within evidence-based breastfeeding support.

Respect for maternal autonomy emerged as another important facilitator of BFHI implementation. Mothers consistently appreciated healthcare providers who engaged them in shared decision-making, explored their breastfeeding goals, and respected their feeding preferences without coercion or judgment. These findings reflect the broader principles underpinning Baby-Friendly care, which emphasize informed decision-making, respectful communication, and individualized support rather than prescriptive approaches to infant feeding [52]. Such relational practices are increasingly recognized as essential components of high-quality maternity care and person-centred health services.

Participants also described early hospital discharge as an important contextual challenge affecting breastfeeding preparedness. Although early discharge reflects broader healthcare system pressures, many mothers reported feeling physically exhausted and emotionally overwhelmed when receiving breastfeeding education shortly before leaving the hospital. Information overload reduced knowledge retention and limited opportunities for hands-on practice during a critical learning period. Similar observations have been reported internationally, where shortened postpartum stays increase reliance on community follow-up services while reducing opportunities to establish breastfeeding confidence before discharge [33].

Cultural expectations and social circumstances further influenced breastfeeding experiences. Participants described how immigration, absence of extended family support, caregiving responsibilities, and early return to work affected breastfeeding continuation despite strong breastfeeding intentions. These findings reinforce socioecological understandings of breastfeeding, demonstrating that maternal behaviours are influenced not only by healthcare services but also by family systems, workplace policies, social norms, and broader structural determinants [20,21,22]. Consequently, successful BFHI implementation requires interventions that acknowledge these wider contextual influences rather than focusing exclusively on hospital-based clinical practices.

Collectively, these findings suggest that breastfeeding support should be viewed as both a clinical and psychosocial intervention. These findings suggest that successful BFHI implementation depends not only on fidelity to the Ten Steps but also on the health system’s capacity to deliver person-centred, psychosocially responsive care. Integrating breastfeeding support with maternal mental health services, culturally responsive care, family-centred education, and broader social support systems may strengthen implementation fidelity while improving breastfeeding sustainability beyond the hospital setting.

### 4.5. Structural and Societal Factors Shaping BFHI Scale-Up (Theme 5)

The final theme illustrates that many barriers to BFHI implementation originate upstream from individual healthcare providers and healthcare facilities. Participants identified inadequate physical infrastructure, geographic inequities, policy gaps, workforce distribution challenges, limited surveillance systems, and the pervasive normalization of formula feeding as structural conditions that constrained implementation efforts. These findings position BFHI implementation as fundamentally dependent upon broader health system capacity rather than solely on provider behavior.

Several participants described hospital environments that were poorly aligned with BFHI principles. Overcrowded postpartum rooms, limited privacy, and infrastructure that restricted rooming-in constrained implementation of baby-friendly practices [6]. While providers demonstrated considerable flexibility in adapting existing environments, these findings illustrate how infrastructure itself can facilitate or constrain implementation regardless of staff commitment.

Geographic inequities represented another major implementation challenge. Families living in rural and northern Saskatchewan frequently encountered transportation barriers, workforce shortages, travel costs, and reduced access to specialized lactation services. Similar disparities have been documented throughout Canada, where rural populations continue to experience inequitable access to maternity and breastfeeding services [53]. These findings suggest that equitable BFHI implementation requires differentiated service delivery models, including expanded telelactation services, strengthened regional referral networks, and targeted investments in rural maternal-child health infrastructure.

Participants also described formula feeding as socially normalized within both healthcare and community settings. Easy access to formula samples, commercial marketing, and inconsistent clinical messaging sometimes undermined breastfeeding intentions even among highly motivated mothers. These findings underscore the importance of protecting breastfeeding from commercial and clinical influences that may undermine informed feeding decisions. Previous evidence has consistently shown that commercial promotion of infant formula influences parental feeding decisions and weakens breastfeeding promotion efforts [6,35,54,55]. The persistence of these influences suggests that effective BFHI implementation requires continued organizational vigilance to ensure adherence to the International Code alongside broader public education initiatives.

Participants additionally identified important weaknesses in provincial governance systems, including inconsistent breastfeeding surveillance, limited monitoring of implementation progress, and unclear organizational accountability. These findings extend beyond traditional discussions of BFHI implementation by emphasizing the importance of health system intelligence. Without standardized provincial indicators, healthcare organizations are limited in their ability to evaluate implementation fidelity, identify performance gaps, and monitor quality improvement efforts. Similar concerns have been identified internationally, where sustainable BFHI implementation has been linked to robust monitoring systems, routine audits, and continuous quality improvement mechanisms [6,33,56]. Taken together, these findings suggest that successful BFHI scale-up requires upstream policy action alongside organizational change. The findings suggest that infrastructure, workforce planning, surveillance systems, and equitable service delivery should be considered alongside clinical education in future BFHI implementation planning and evaluation.

### 4.6. Implications for Policy and Practice

This study identifies several priorities for strengthening BFHI implementation and scale-up within Saskatchewan. First, implementation strategies should move beyond a primary focus on staff education and adopt a systems-oriented approach that strengthens leadership continuity, organizational governance, workforce planning, and accountability. Provincial health authorities and healthcare organizations could establish permanent interdisciplinary BFHI or breastfeeding governance committees with defined responsibilities, implementation targets, and reporting mechanisms. Embedding BFHI responsibilities within organizational quality-improvement plans and leadership accountability structures could reduce vulnerability to administrative turnover and ensure that breastfeeding priorities remain institutional rather than dependent on individual champions.

Second, investments in workforce capacity remain essential. Health authorities could establish minimum breastfeeding competency requirements for healthcare professionals involved in maternity and newborn care and incorporate these competencies into staff orientation, annual education, and ongoing professional development. Increasing access to funded IBCLC certification, protected time for breastfeeding education and mentorship, and interdisciplinary competency development could improve the consistency of breastfeeding support across settings. Competency-based education should extend beyond knowledge acquisition to include supervised clinical practice, mentorship, and regular competency assessment aligned with BFHI standards. Standardized clinical pathways for breastfeeding assessment and medically indicated supplementation could further reduce variation in practice across providers, shifts, and facilities.

Third, strengthening continuity of care across hospital, public health, and community services should remain a central implementation priority. Provincial and regional health authorities could establish standardized hospital-to-community referral pathways, with clear referral criteria, defined follow-up timelines, and mechanisms for communicating referral outcomes between services. Electronic referral systems and shared documentation could improve coordination among maternity units, public health nursing, lactation consultants, primary care providers, and community breastfeeding programs. Expanding public health follow-up and community-based breastfeeding services, together with telelactation and other virtual support models, could improve access for rural and northern populations where specialized services are limited.

Finally, provincial implementation would benefit from a standardized breastfeeding monitoring and accountability system. The provincial health system could establish a core set of indicators assessing adherence to BFHI practices, breastfeeding outcomes, supplementation practices, referral and follow-up, and implementation fidelity across healthcare facilities. Routine collection, audit, and reporting of these indicators would allow organizations to identify gaps, compare implementation across settings, and evaluate the effectiveness of quality-improvement initiatives. Where feasible, monitoring data should be examined by geographic location to identify inequities in access and implementation, particularly for rural and northern communities. Together, these proposed mechanisms provide potential directions for moving BFHI scale-up beyond reliance on individual commitment toward a more coordinated and measurable health-system approach, which should be evaluated in future research.

### 4.7. Strengths and Limitations

This study provides one of the first comprehensive qualitative explorations of BFHI implementation and scale-up within Saskatchewan. A major strength is the inclusion of multiple stakeholder perspectives, including mothers with lived breastfeeding experience and healthcare professionals working across maternal-child health, public health, nutrition, lactation support, and community settings. Some participants brought both maternal lived experience and professional expertise, providing a unique perspective on breastfeeding support from both sides of the healthcare relationship. Participants also represented urban, regional, smaller, Indigenous, and remote communities across Saskatchewan, providing perspectives from diverse geographic and community contexts. The inclusion of these perspectives enabled triangulation across participants and supported a systems-level understanding of factors influencing BFHI implementation.

The application of Bronfenbrenner’s socioecological model further strengthened interpretation by situating BFHI implementation within interacting individual, interpersonal, organizational, community, and policy contexts rather than focusing exclusively on provider behaviour. While the model supported a multi-level understanding of the Saskatchewan context, it does not fully capture implementation-specific processes such as implementation readiness, strategy selection, fidelity, or sustainability. Future research could complement this socioecological perspective with implementation-science approaches to further examine how BFHI implementation can be effectively adopted, sustained, and scaled across diverse healthcare settings.

Several limitations should also be considered. First, although the study included multiple stakeholder perspectives, these perspectives were not equally distributed across mutually exclusive participant groups. The sample included 17 mothers and 15 healthcare professionals, with six participants occupying both roles. Participants with dual maternal and professional identities may have brought valuable insights arising from the intersection of lived experience and clinical expertise; however, their perspectives may also have influenced the relative prominence of maternal and professional viewpoints within the analysis. Although the analysis considered perspectives across participant roles, the overlap between these groups should be considered when interpreting the findings. Future research could seek more balanced representation of distinct stakeholder groups, including mothers, frontline healthcare providers, community-based providers, and health system decision-makers.

Second, although participants included individuals from diverse communities across Saskatchewan, representation of some stakeholder and geographic groups was limited. The sample included participants from smaller, remote, and Indigenous communities; however, the number of participants from these communities was not sufficient to support detailed subgroup-specific analysis or to capture the full diversity of Indigenous and geographically remote experiences. The findings therefore should not be interpreted as representing the experiences or priorities of all Indigenous communities or all northern and remote populations in Saskatchewan. Future research should involve larger and more intentionally community-engaged samples from Indigenous, northern, rural, and remote communities and should be conducted in partnership with relevant Indigenous communities and organizations to ensure that local priorities, cultural contexts, and community-defined approaches to breastfeeding support are appropriately reflected.

Third, the perspectives of senior health system decision-makers and policy-level leaders were limited. Although participants provided important insights into leadership, governance, organizational policies, and system-level barriers, the study did not capture the full range of perspectives of individuals responsible for provincial policy development, resource allocation, implementation oversight, and organizational accountability. Their perspectives may provide additional insight into the feasibility, prioritization, and sustainability of strategies for BFHI scale-up. Future studies should therefore include a broader range of decision-makers and policy stakeholders.

The study was also conducted within a single Canadian province and reflects the organizational, geographic, and policy context of Saskatchewan. Although many findings may be transferable to jurisdictions facing similar implementation challenges, they should not be interpreted as representative of all Canadian healthcare settings. Participation was voluntary, and individuals with strong interest in breastfeeding or relevant experience may have been more likely to participate, introducing the possibility of self-selection bias. As with qualitative research generally, the findings are intended to provide in-depth contextual understanding rather than statistical generalizability. Nevertheless, the inclusion of multiple stakeholder perspectives, diverse geographic contexts, and participants with both lived and professional experience provides a valuable systems-level understanding of the conditions influencing BFHI implementation and scale-up in Saskatchewan.

## 5. Conclusions

Within the Saskatchewan context, this study provides context-specific qualitative evidence that BFHI implementation and scale-up are shaped by interactions among organizational capacity, workforce readiness, continuity of care, maternal experiences, and broader structural conditions. The findings indicate that implementation challenges extend beyond individual provider practices and are influenced by the capacity of the wider health system to sustain coordinated, equitable, and consistent breastfeeding support. These findings can inform future BFHI planning, policy development, and implementation efforts in Saskatchewan, particularly those focused on leadership continuity, workforce capacity, integrated care pathways, equitable access to breastfeeding services, and system-level monitoring and accountability. However, this study did not evaluate the feasibility or effectiveness of specific system-level strategies. Future research should examine how identified priorities can be translated into contextually appropriate implementation strategies and assess their feasibility, sustainability, and effectiveness across diverse healthcare and geographic settings.

## Figures and Tables

**Table 1:** Qualitative Data Analysis Process.

Initial Codes	Categories	Sub-Themes	Final Themes
Supportive manager; breastfeeding champion; leadership encouragement; organizational prioritization of breastfeeding	Organizational commitment and leadership support	Leadership engagement and organizational commitment	Theme # 1: Leadership and Governance as Foundations for BFHI Implementation
Breastfeeding advisory committee; interdisciplinary collaboration; provincial breastfeeding guidelines; standardized protocols	Governance mechanisms and practice alignment	Governance structures and standardized approaches	
Leadership changes; loss of champions; shifting priorities; reduced institutional memory	Challenges maintaining implementation momentum	Leadership continuity and sustainability challenges	
Hands-on breastfeeding assistance; latch support; positioning guidance; knowledgeable nurses	Provider competency and individualized breastfeeding care	Skilled and responsive breastfeeding support	Theme # 2: Workforce Capacity and Clinical Practice Consistency as Determinants of Care Delivery
Staffing shortages; workload pressures; competing clinical priorities; limited time for breastfeeding support	Workforce constraints affecting care delivery	Staffing pressures and inconsistent breastfeeding practices	
Conflicting professional recommendations; supplementation discussions; provider variation; limited lactation certification	Variability in clinical expertise and decision-making	Professional practice variation and lactation capacity challenges	
Maternal visiting programs; public health follow-up; lactation referrals; peer breastfeeding groups	Community-based breastfeeding support systems	Public health and community support as facilitators	Theme # 3: Continuity of Breastfeeding Support Across Care Settings
Delayed referrals; limited follow-up frequency; fragmented services; unmet breastfeeding needs	Gaps in care transitions	Challenges in continuity and referral pathways	
Postpartum vulnerability; emotional distress; need for compassion; maternal confidence	Maternal psychological wellbeing and supportive care	Mental health and respectful breastfeeding support	Theme # 4: Breastfeeding as a Psychosocially Embedded Maternal Experience
Limited breastfeeding education; early discharge; insufficient preparation; information overload	Breastfeeding knowledge and readiness	Education needs during early postpartum transition	
Cultural expectations; family support differences; workplace demands; early return to work	Social determinants influencing breastfeeding	Cultural, social, and workplace influences	
Hospital space limitations; privacy concerns; barriers to rooming-in	Healthcare environment constraints	Physical environments influencing BFHI practices	Theme # 5: Structural and Societal Factors Shaping BFHI Scale-up
Rural transportation challenges; distance to services; financial barriers	Inequitable access to breastfeeding support	Geographic and socioeconomic barriers	
Formula marketing; normalization of formula feeding; unsolicited supplementation	Sociocultural influences on infant feeding	Formula normalization and competing feeding messages	
Lack of breastfeeding surveillance; limited provincial data; unclear accountability	Policy and system-level gaps	Data limitations and fragmented accountability	

**Table 2:** Demographic Characteristics of Study Participants (*n* = 26).

Characteristic	*n*
Participant group (*n* = 26)	
Mothers only	11
Mothers who were also healthcare professionals	6
Healthcare professionals without maternal experience	9
Healthcare professional status (*n* = 15)	
● Registered nurses/licensed practical nurses	9
● Registered nurses/licensed practical nurses with IBCLC certification	4
● Pediatric dietitians/public health nutritionists	2
Healthcare Practice Settings (*n* = 15)	
● Hospital-based maternal care (prenatal, labor & birth, and postnatal units)	3
● Public health and community visiting programs	4
● Neonatal Intensive Care Unit (NICU)	2
● Acute care hospital settings	3
● Nutrition Services (hospital and community)	2
● Private lactation practice (community-based)	1
Participants’ Geographic location in Saskatchewan, Canada (*n* = 26)	
● Moose Jaw	8
● Regina	6
● Saskatoon	5
● Prince Albert	2
● La Ronge	1
● Yorkton	1
● White City	1
● Balgonie	1
● Chaplin/Central Butte	1
Infant-related experiences among mothers (*n* = 17)	
● No reported infant clinical concerns	8
● Infant with tongue-tie/lip-tie	4
● Infant with jaundice	3
● Infant admitted to NICU	2

**Table 3:** Summary of Themes, Barriers, Facilitators, Socioecological Levels, and Participant Perspectives.

Theme	Key Facilitators	Key Barriers	Socioecological Level(s)	Participant Perspectives
1. Leadership and Governance as Foundations for BFHI Implementation	Supportive leadership; breastfeeding champions; organizational prioritization; breastfeeding recognition initiatives; provincial guidelines; breastfeeding committees; interdisciplinary collaboration; standardized protocols	Leadership turnover; loss of breastfeeding champions; shifting institutional priorities; reduced organizational memory; dependence on individual leaders	Organizational; Health system	Mothers who were healthcare professionals; healthcare professionals, including IBCLCs
2. Workforce Capacity, Competency and Practice Consistency as Determinants of Care Delivery	Knowledgeable and skilled providers; hands-on latch and positioning support; individualized breastfeeding assistance; provider commitment	Staffing shortages; workload pressures; competing clinical priorities; inconsistent provider recommendations; professional practice variation; limited lactation expertise; financial barriers to IBCLC certification	Individual; Interpersonal; Organizational; Health system	Mothers; mothers who were healthcare professionals; healthcare professionals, including IBCLCs
3. Continuity of Breastfeeding Support Across Care Settings	Public health follow-up; maternal visiting programs; home visits; lactation referrals; peer breastfeeding groups; community programs; access to breast pumps	Delayed referrals; limited follow-up frequency; fragmented services; inconsistent referral pathways; gaps between hospital and community services	Interpersonal; Organizational; Community; Health system	Mothers; mothers who were healthcare professionals; healthcare professionals
4. Breastfeeding as a Psychosocially Embedded Maternal Experience	Compassionate and respectful care; maternal choice and preferences; breastfeeding education; emotional support; individualized communication	Maternal emotional vulnerability; insufficient breastfeeding education; information overload; early postpartum discharge; cultural differences; limited family support; workplace demands and early return to work	Individual; Interpersonal; Community; Structural	Mothers; mothers who were healthcare professionals; healthcare professionals
5. Structural and Societal Factors Shaping BFHI Scale-up	Breastfeeding-supportive environments; access to community resources; shared decision-making	Crowded hospital environments; limited privacy; barriers to rooming-in; geographic and transportation barriers; financial barriers; formula normalization and marketing; limited breastfeeding surveillance; fragmented accountability; limited access to lactation services	Organizational; Community; Health system; Structural	Mothers; mothers who were healthcare professionals; healthcare professionals

## Data Availability

The data contain sensitive information about study participants, and based on the informed consent process, data containing participant information cannot be shared publicly.

## Abbreviations

The following abbreviations are used in this manuscript:

BFHI	Baby-Friendly Hospital Initiative
IBCLC	International Board-Certified Lactation Consultant
NICU	Neonatal Intensive Care Unit
WHO	World Health Organization
